# A 45° fibular tunnel promotes better graft healing than a 30° tunnel in patients with chronic lateral ankle instability undergoing anatomic reconstruction

**DOI:** 10.1002/jeo2.70980

**Published:** 2026-11-09

**Authors:** Haoxuan Liu, Yuqing Zhao, Tong Su, Xing Xie, Qinwei Guo, Chen Jiao, Yanbin Pi, Dong Jiang

**Affiliations:** ^1^ Department of Sports Medicine Peking University Third Hospital, Institute of Sports Medicine of Peking University, Beijing Key Laboratory of Sports Injuries, Engineering Research Center of Sports Trauma Treatment Technology and Devices, Ministry of Education Beijing China; ^2^ Department of Radiology Peking University Third Hospital Beijing China

**Keywords:** anatomic reconstruction, anterior talofibular ligament, chronic lateral ankle instability, graft healing, MRI

## Abstract

**Purpose:**

To investigate the effect of different fibular tunnel directions on the postoperative graft healing and patient‐reported outcomes of chronic lateral ankle instability (CLAI) in patients following anatomic reconstruction using gracilis tendon autograft.

**Methods:**

A total of 72 CLAI patients following anatomic reconstruction at our institution from June 2021 to May 2022 were retrospectively reviewed and were included in the 30° fibular tunnel group and the 45° fibular tunnel group based on the surgical records. Patients were further divided into two groups based on whether the calcaneofibular ligament was reconstructed, and 1:1 propensity score matching was performed separately in the two groups. The signal‐to‐noise quotient (SNQ) was measured through magnetic resonance imaging scans at 12 months postoperatively. The graft bend angle (GBA) was measured through computed tomography scans at 1 day postoperatively. The Visual Analogue Scale (VAS), Tegner Activity Level Scale and Karlsson–Peterson ankle score (Karlsson score) were also assessed at the 12‐month and 24‐month follow‐ups.

**Results:**

A total of 31 patients were included in each group. The anterior talofibular ligament (ATFL) GBA on the fibular side was significantly smaller in the 45° fibular tunnel group than the 30° group (38.6 ± 11.2 vs. 52.5 ± 11.4, *p* < 0.001). The 45° fibular tunnel group exhibited significantly lower SNQ values than the 30° group, both for the 1/3 at the fibular side (10.6 ± 2.7 vs. 13.1 ± 2.9, *p* = 0.001) and for the entire ATFL graft (11.2 ± 2.9 vs. 13.3 ± 2.8, *p* = 0.011). There was no significant difference in clinical outcomes between the 45° fibular tunnel group and the 30° group.

**Conclusion:**

Compared with the 30° fibular bone tunnel, the 45° tunnel promotes graft healing in CLAI patients following anatomic reconstruction using an autograft.

**Level of Evidence:**

Level III, retrospective study.

**Abbreviations**
ACL: anterior cruciate ligament
ATFL: anterior talofibular ligament
BMI: body mass index
CFL: calcaneofibular ligament
CLAI: chronic lateral ankle instability
CT: computed tomography
GBA: graft bend angle
MPR: multi‐planar reconstruction
MRI: magnetic resonance imaging
SNQ: signal‐to‐noise quotient
VAS: visual analogue scale

## INTRODUCTION

Lateral ankle sprain is one of the most prevalent sports injuries [33]. Due to injuries to the anterior talofibular ligament (ATFL) and calcaneofibular ligament (CFL), 20%–30% of individuals can suffer from chronic lateral ankle instability (CLAI), which is characterized by recurrent sprain, sense of giving‐way, persistent pain, deficits of postural control and muscle strength [29]. Once rigorous conservative approaches failed, surgical intervention is considered [2]. The current gold standard surgical treatment for CLAI is the modified Broström procedure [30]. However, when the patients were confirmed with with irreparable ATFL and CFL or isolate ATFL ligament remnant intraoperatively, anatomic reconstruction with autograft or allograft is usually considered as the preferred alternative to provide better stability [16, 22, 35].

The direction of the fibular bone tunnels represents a critical yet controversial aspect of anatomical reconstruction techniques [9]. To date, previous studies have primarily compared the intraoperative bone and tendon damage caused by different fibular tunnel directions [10, 14, 18]. However, no studies have yet investigated whether different fibular tunnel directions affect patients' postoperative clinical outcomes. A relevant concept for understanding this potential effect is the graft bend angle (GBA) in anterior cruciate ligament (ACL) reconstruction [27, 32]. Several previous studies have shown that a larger GBA may lead to greater compression and shear forces between the graft and the tunnel aperture [19, 23], resulting in delayed postoperative graft maturation [5, 26]. Similarly, although fixation techniques differ, after ankle ligament reconstruction, this mechanism may also theoretically exist.

Therefore, the current study was designed to investigate the effect of different fibular tunnel directions on the postoperative graft healing of CLAI patients following anatomic reconstruction using gracilis tendon autograft. It was hypothesized that a 45° fibular tunnel can promote graft healing in CLAI patients following anatomic reconstruction using an autograft.

## METHODS

### Patient enrolment

This was a single‐centre retrospective cohort study approved by our institutional review board. The CLAI patients following anatomic reconstruction using gracilis tendon autograft was enroled when the patient met all of the following criteria [34]: (1) a traumatic history of at least one significant ankle sprain, and the most recent ankle sprain occurred >3 months prior to study participation; (2) persistent clinical symptoms including swelling, ‘giving way’ sense, feeling of instability or recurrent sprain for at least 3 months; (3) Grade III anterior drawer test and varus stress test confirmed by physical examinations of two experienced doctors, and chronic ATFL and CFL injury or isolate ATFL injury confirmed by magnetic resonance imaging (MRI); (4) failure of rigorous conservative treatment including muscle strength training and balance training of at least 6 months supervised by a physical therapist with more than 4 years of experience; (5) arthroscopic exploration using a probe confirmed the ATFL and CFL or isolate ATFL had poor tension or was missing that could not be repaired and underwent anatomic reconstruction using gracilis tendon autograft of ATFL and CFL or isolate ATFL. Patients were excluded if they had the following criteria: (1) the patients had undergone combined microfracture since the postoperative protocol differs, bilateral ankle instability, subtle cavus foot deformity or forefoot‐driven hindfoot varus, or other lower limb musculoskeletal disorders; (2) combined central and peripheral neuromuscular disease; (3) had progressing or early‐stage ankle arthritis (Takakura arthritis classification ≥ stage 3); (4) unwilling to participate.

A list of patients who underwent anatomic reconstruction at our institution from June 2021 to May 2022 was retrospectively obtained. At our institution, patients routinely complete preoperative patient‐reported outcomes scoring questionnaires 1 day before surgery, and consent to be followed up postoperatively was signed. Before the 12‐month follow‐up, the researchers obtained patients' consent for study inclusion via telephone follow‐up, and patients returned to the institution at 12 months postoperatively for an in‐person evaluation including MRI scans, after which written informed consent was signed. At the 24‐month follow‐up, the questionnaires were sent to patients who were willing to engage. 72 patients who completed the entire follow‐up protocol were included for final analysis and were assigned to the 30° fibular tunnel group and the 45° fibular tunnel group based on surgical records and further confirmed by the tunnel direction measured on computed tomography (CT) scans acquired at 1 day postoperatively.

Since the reconstruction of the CFL or not influences the placement of the fibular tunnel, the 72 patients were initially divided into two groups based on whether CFL reconstruction was performed [25]. Then, to minimize confounding by other potential factors, a 1:1 propensity score matching was conducted within the two groups separately based on age at surgery, gender, body mass index (BMI), post‐injury duration, pre‐injury Tegner score, the presence of os subfibulare, the presence of osteochondral lesion of the talus and follow‐up duration. As shown in Figure 1, according to the matching results, 31 patients were included in each group.

**Figure 1:**
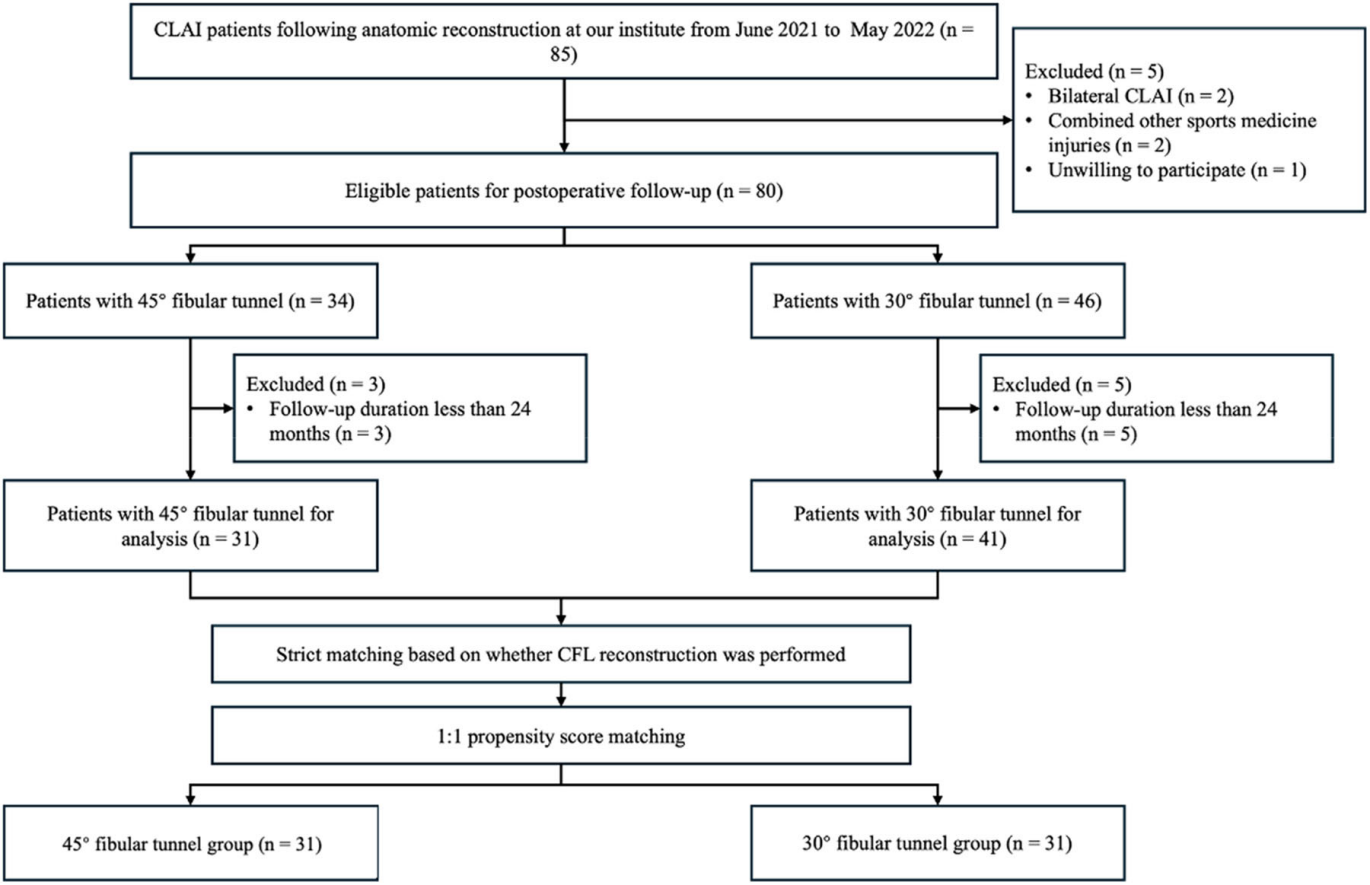
Patient enrolment. CFL, calcaneofibular ligament; CLAI, chronic lateral ankle instability.

### Surgical procedure and postoperative rehabilitation

All anatomic reconstructions using an autograft for CLAI patients were performed according to previous studies [24, 25]. Patients underwent either spinal or general anaesthesia and were placed in a supine position, and standard anteromedial and anterolateral portals were established for diagnostic arthroscopy using a 2.9 mm 30° arthroscopic system (Smith & Nephew) to assess and manage intra‐articular pathologies. The ipsilateral gracilis tendon was then harvested and doubled folded at the midpoint of the graft, and a 4–5 cm oblique incision was made anteroinferior to the fibula. When the CFL remnant exhibited excellent quality or remained intact, it was either repaired or left untreated. Following debridement of the ATFL footprints, two bone tunnels were completed and drilled through the fibula and the talus for isolated ATFL reconstruction. If the CFL remnant quality was poor, a Y‐shaped graft was prepared. A fibular tunnel was completed and drilled at the common insertion footprints of the ATFL and CFL through the fibula, while talar and calcaneal tunnels were drilled and completed through the talus and calcaneus at the insertion footprints of the ATFL and CFL. The tendon was introduced first into the fibular tunnel and then into the talar and calcaneal tunnels and was fixed by Polyether Ether Ketone interference screws in neutral ankle position, when the ankle was at 10° of plantar flexion, with 0° of both inversion and eversion. Figure 2 is the schematic illustration of the bone tunnel direction.

**Figure 2:**
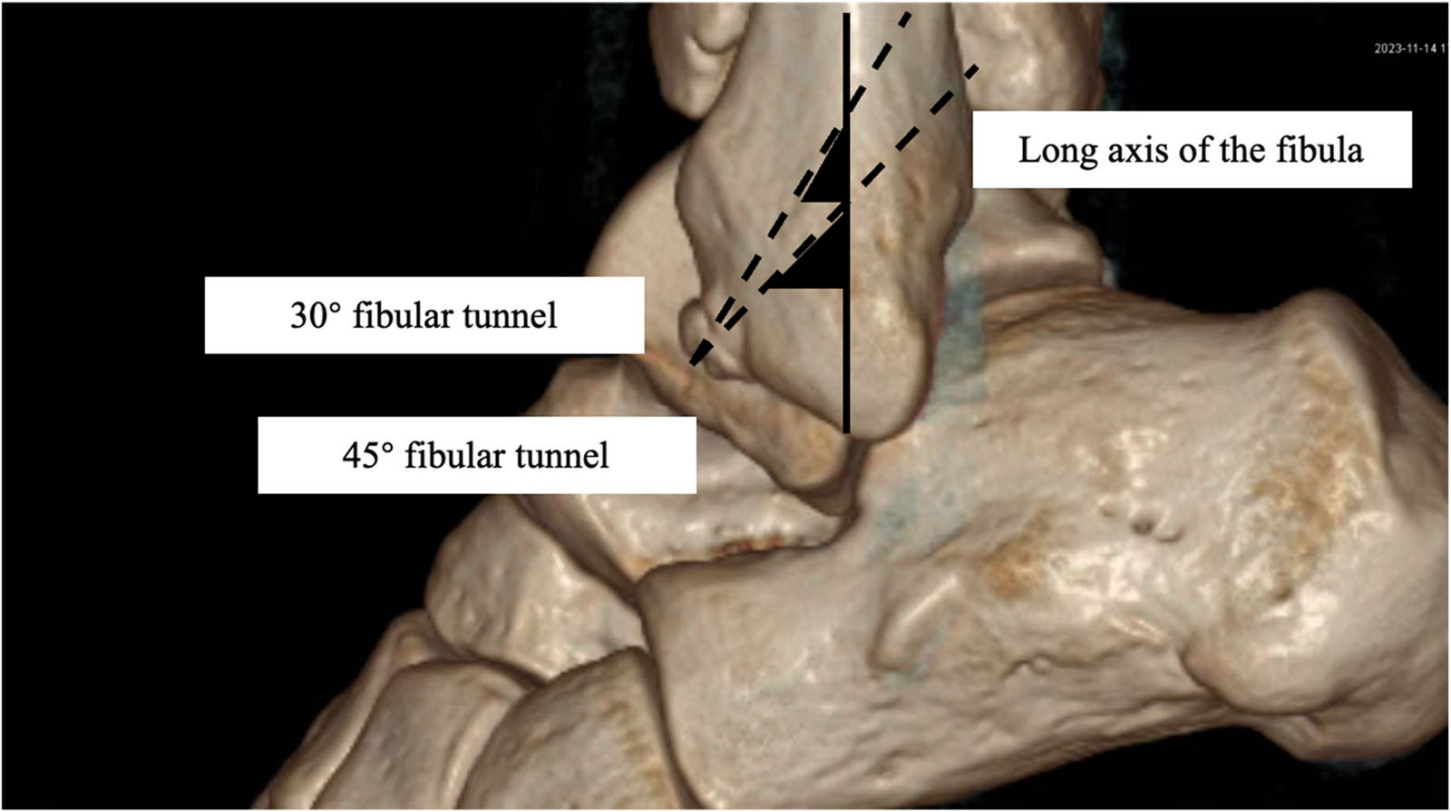
Schematic illustration of the bone tunnel direction.

After the surgery, the ankle was immobilized with a short leg cast for the first 2 weeks. Partial weight‐bearing was allowed at Weeks 2–4 postoperatively and gradually transitioned to full weight‐bearing with a walking brace. From Week 6, weight‐bearing muscle strength exercise was allowed, and dynamic balance exercise started at Week 7. All the patients returned to full weight‐bearing walking and sport based on their tolerance. Then, patients were able to resume their daily activities and engage in sports that fell within their physical capabilities.

### MRI measurement

A 3.0 T MRI scanner (PRISMA; Siemens) was used to acquire the MRI scans at 12 months postoperatively. The ankle was maintained in the neutral position. All images were reformatted, and measurements were conducted using uPACS R001 (United Imaging). The signal‐to‐noise quotient (SNQ) values of the reconstructed ligaments were measured in the fat‐suppressed proton‐weighted magnetic resonance images, and the axial magnetic resonance image that best showed the entire length of the reconstructed ligament was selected [8, 12]. On this layer, the polygon tool was used to delineate the range of the ligament. The average signal value of the ligament was recorded. Then, the reconstructed ligament length was divided into three equal regions, including the fibula 1/3, middle 1/3 and talar 1/3, and the average signal values of the three regions were recorded, respectively. A circular region of interest with a radius of 1 mm was drawn on the outer side of the skin surface of the lateral ankle, and the average value was recorded as the noise value. As shown in Figure 3, the SNQ was calculated as the quotient of the signal value of ATFL and the signal value of the noise.

**Figure 3:**
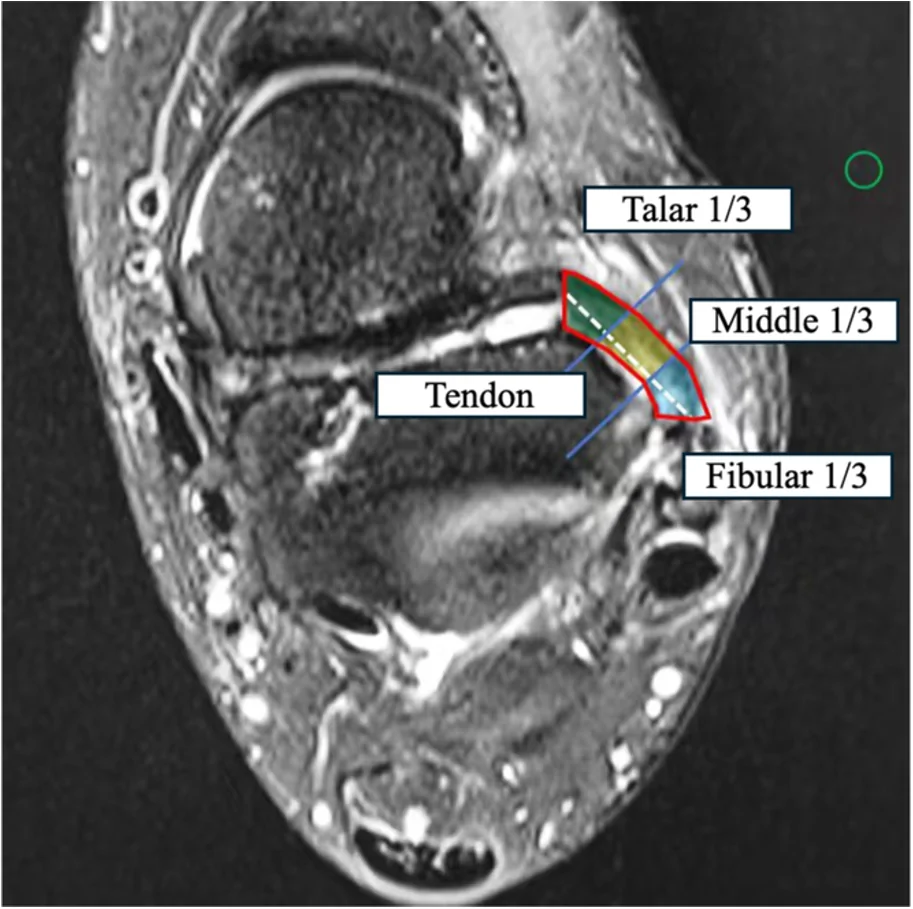
SNQ measurement. SNQ, signal‐to‐noise quotient.

### CT measurement

A multichannel CT scanner (uCT 790; United Imaging) was used to acquire CT scans at Day 1 postoperatively. The ankle was immobilized by a cast in the neutral position. All images were reformatted, and measurements were conducted by AW Server 4.7 (GE Healthcare).

The direction of the bone tunnel was determined by the angle between the tunnel and the long axis of the fibula. The long axis of the fibula was constructed by identifying the centres of two circles on the fibula.

The measurement of GBA was carried out using orthogonal multi‐planar reconstruction (MPR) in thin‐layer images with a layer thickness and spacing of 0.75 mm. The centre point was positioned at the centre of the bone tunnel. Rotate the reconstruction planes so that one of the orthogonal planes is perpendicular to the long axis direction of the bone tunnel to be measured. At this time, the images of the other two orthogonal planes can show the total length of the bone tunnel. Keep the plane perpendicular to the long axis direction of the bone passage unchanged, and adjust the directions of the other planes so that one of them simultaneously shows the long axis of the bone passage and the full length of the reconstructed ligament. As shown in Figure 4, the angle between the bone passage and the ligament shown on this plane was the GBA angle.

**Figure 4:**
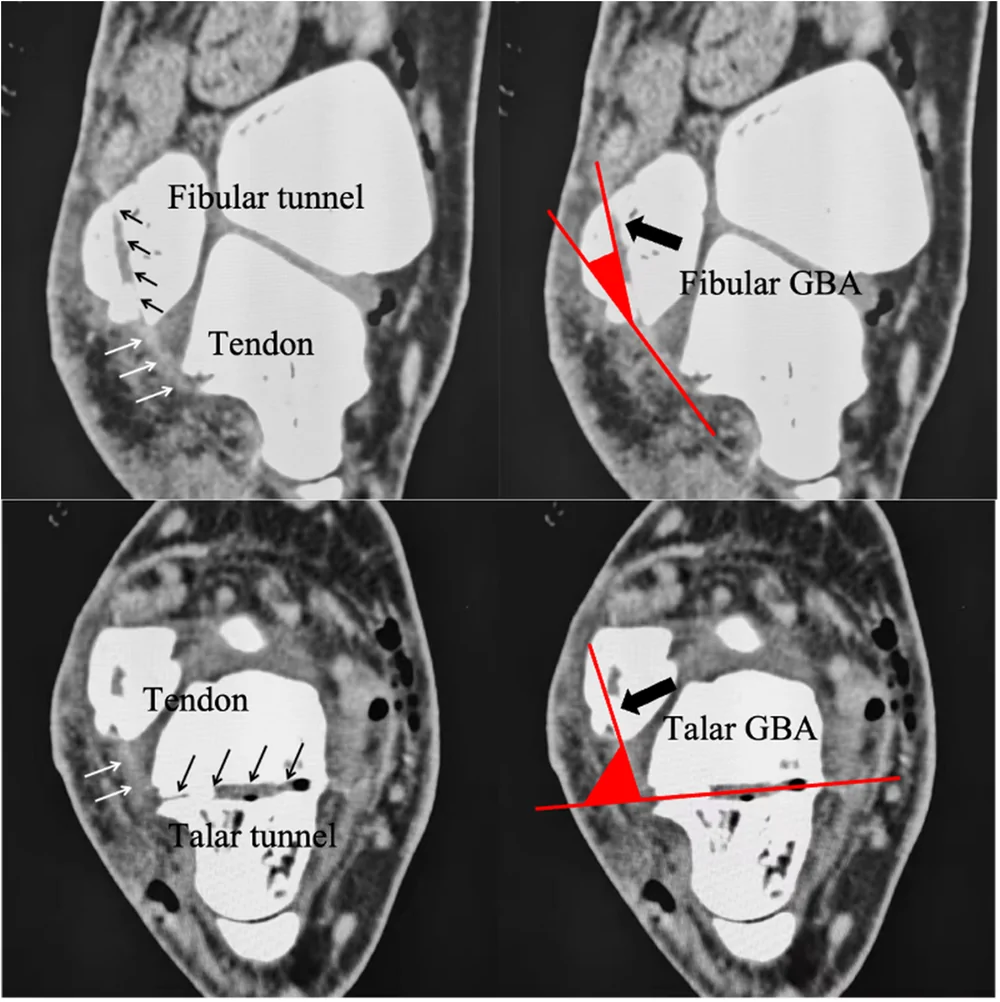
GBA measurement. GBA, graft bend angle.

### Outcome evaluation

Patient demographics, including age at surgery, gender, height, weight, BMI, post‐injury duration and pre‐injury Tegner score were collected from preoperative medical records. Intraoperative findings such as CFL reconstruction, the presence of os subfibulare, the presence of an osteochondral lesion of the talus and the presence of an osteophyte were collected from surgical records.

The primary outcome of the study was the SNQ value of the reconstructed ATFL. Besides, clinical outcomes were assessed preoperatively, 12 months postoperatively and 24 months postoperatively to further investigate the potential effect of tunnel angle on clinical outcomes using the visual analogue scale (VAS score), Tegner Activity Level Scale (Tegner score) and Karlsson–Peterson ankle score (Karlsson score) [1].

### Statistical analysis

PASS Sample Size Software 15 (NCSS, LLC) was used for sample size calculation. Since no previous studies have compared the SNQ value of the ATFL at the fibular 1/3 of the patients between the two groups, the power of the study was calculated post hoc using the actual effect size of 0.918. When an α was set at 0.05, the study achieved an actual power of 0.945.

All the data were analysed by IBM SPSS Statistics 24.0 (SPSS Inc.). The Shapiro–Wilk test was first performed to check for normal distribution of each group. The data were presented as the mean ± SD for continuous variables and frequency (percentage) for categorical data. The Pearson's *χ* ^2^ test, Mann–Whitney *U* test or independent *t* test (two‐sided test and *α* = 0.05) was applied to compare the differences between groups. Differences were considered to be significant at *p* < 0.05.

The inter‐observer reliability and intra‐observer reproducibility of the measurement methods were examined according to previous studies [4]. To evaluate inter‐observer reliability, a musculoskeletal radiologist and a foot and ankle specialist independently performed measurements on the CT and MRI images. Subsequently, the musculoskeletal radiologist performed all final measurements to reduce inter‐observer variability. For blinding, the patient list was randomized, and each patient was assigned a coded number for each measurement session, so that the measurer was blinded to patient identity. To assess intra‐observer reproducibility, the measurements were repeated initially and after a 2‑week interval. The intraclass correlation coefficient (ICC) was then calculated to evaluate the test–retest repeatability of the evaluation methods, which could be rated as excellent (ICC > 0.9), good (0.75 < ICC < 0.9), moderate (0.5 < ICC < 0.75) or poor (ICC < 0.5) [15, 31]. After the ICC tests, the order of the selected patient list was randomized to mitigate potential bias, and one author completed all measurements to reduce inter‐observer variability. The inter‐observer reliability and the intra‐observer reproducibility of the SNQ measurement, tunnel direction measurement and GBA measurement were all rated as excellent (all ICCs > 0.9).

## RESULTS

### Comparison of the patient demographics between the 45° fibular tunnel group and the 30° fibular tunnel group

As shown in Table 1, there was no significant difference in the patient demographics between the 45° fibular tunnel group and the 30° fibular tunnel group.

**Table 1:** Comparison of the patient demographics between the 45° fibular tunnel group and 30° fibular tunnel group.

	45° fibular tunnel group (*n* = 31)	30° fibular tunnel group (*n* = 31)	*p* value
Gender, male/female (%)	19 (61.3)/12 (38.7)	18 (58.1)/13 (41.9)	0.796
Side, right/left (%)	14 (45.2)/17(54.8)	15 (48.4)/16 (51.6)	0.799
Age at surgery, years	31.0 ± 8.1	29.0 ± 8.2	0.308
BMI, kg/m^2^	25.1 ± 3.6	24.4 ± 3.8	0.428
Beighton score	3.3 ± 3.0	3.6 ± 2.9	0.283
Post‐injury duration, months	29.9 ± 35.7	31.9 ± 41.7	0.608
Pre‐injury Tegner score	5.9 ± 0.7	5.6 ± 0.6	0.422
CFL status			
Intact CFL, yes/no, (%)	5 (16.1)/26 (83.9)	7 (22.6)/24 (77.4)	
CFL repair, yes/no, (%)	16 (51.6)/15 (48.4)	14 (45.2)/17 (54.8)	
CFL reconstruction, yes/no, (%)	10 (32.3)/21 (67.7)	10 (32.3)/21 (67.7)	
Os subfibulare, yes/no, (%)	9 (29.0)/22 (71.0)	8 (25.8)/23 (74.2)	0.776
Osteochondral lesion of the talus, yes/no, (%)	12 (38.7)/19 (61.3)	11 (35.5)/20 (64.5)	0.793
Osteophyte, yes/no, (%)	11 (35.5)/20 (64.5)	10 (32.3)/21 (67.7)	0.788

Abbreviations: BMI, body mass index; CFL, calcaneofibular ligament.

### Comparison of the MRI and CT measurements between the 45° fibular tunnel group and the 30° fibular tunnel group

As shown in Table 2, the ATFL GBA on the fibular side was significantly smaller in the 45° group than in the 30° group (38.6 ± 11.2 vs. 52.5 ± 11.4°, *p* < 0.001). The SNQ value of the fibular 1/3 (10.6 ± 2.7 vs. 13.1 ± 2.9, *p* = 0.001) and entire ATFL graft (11.2 ± 2.9 vs. 13.3 ± 2.8, *p* = 0.011) was significantly lower in the 45° fibular tunnel group compared with the 30° group.

**Table 2:** Comparison of the CT and MRI measurements between the 45° fibular tunnel group and the 30° fibular tunnel group.

	45° fibular tunnel group (*n* = 31)	30° fibular tunnel group (*n* = 31)	*p* value
Fibular tunnel direction, °	45.3 ± 3.5	31.2 ± 4.1	<0.001*
ATFL GBA			
Fibular side, °	38.6 ± 11.2	52.5 ± 11.4	<0.001*
Talar side, °	67.2 ± 12.9	65.3 ± 13.4	0.611
ATFL SNQ			
Entire graft	11.2 ± 2.9	13.3 ± 2.8	0.011*
Fibular 1/3	10.6 ± 2.7	13.1 ± 2.9	0.001*
Middle 1/3	10.9 ± 4.0	12.6 ± 3.4	0.081
Talar 1/3	12.6 ± 4.2	13.4 ± 4.1	1.000

Abbreviations: ATFL, anterior talofibular ligament; CT, computed tomography; GBA, graft bend angle; MRI, magnetic resonance imaging; SNQ, signal‐to‐noise quotient.

* Significant difference between groups.

### Comparison of the clinical outcomes between the 45° fibular tunnel group and the 30° fibular tunnel group

As shown in Table 3, there was no significant difference in the clinical outcomes between the 45° fibular tunnel group and the 30° fibular tunnel group.

**Table 3:** Comparison of the clinical outcomes between the 45° fibular tunnel group and the 30° fibular tunnel group.

	45° fibular tunnel group (*n* = 31)	30° fibular tunnel group (*n* = 31)	*p* value
VAS score			
Preoperative	3.4 ± 1.1	3.5 ± 1.1	0.655
Postoperative 12 m	0.6 ± 0.8	0.7 ± 0.8	0.652
Postoperative 24 m	0.3 ± 0.8	0.3 ± 0.6	0.748
Tegner score			
Preoperative	3.5 ± 0.8	3.4 ± 0.8	0.748
Postoperative 12 m	5.0 ± 0.7	5.3 ± 1.0	0.226
Postoperative 24 m	5.7 ± 0.9	5.6 ± 1.0	0.761
Karlsson score			
Preoperative	65.3 ± 7.4	66.9 ± 7.8	0.398
Postoperative 12 m	93.7 ± 3.7	93.6 ± 1.6	0.894
Postoperative 24 m	95.8 ± 4.9	95.3 ± 3.5	0.780
Re‐sprain, yes/no, (%)	1 (3.2)/30 (96.8)	2 (6.5)/29 (93.5)	1.000
Return to recreational sports, yes/no, (%)	29 (93.5)/2 (6.5)	29 (93.5)/2 (6.5)	1.000
Return to pre‐injury sports, yes/no, (%)	23 (74.2)/8 (25.8)	22 (71.0)/9 (29.0)	0.776
Other surgical complications, yes/no, (%)	5 (16.1)/26(83.9)	4 (12.9)/27 (87.1)	0.718

Abbreviation: VAS, visual analogue scale.

## DISCUSSION

The main finding of our study was that, compared with a 30° fibular bone tunnel, a 45° tunnel can lead to lower ATFL SNQ in CLAI patients following anatomic reconstruction using an autograft. To our knowledge, this is the first study to investigate factors affecting tendon ligamentization of CLAI patients following anatomic reconstruction.

The ATFL GBA on the fibular side was significantly smaller in the 45° group than in the 30° group, and the SNQ value of the fibular 1/3 and entire ATFL graft was significantly lower in the 45° fibular tunnel group compared with the 30° group. Several previous studies have primarily compared the intraoperative bone and tendon damage caused by different fibular tunnel directions [10, 14, 18]. Michels et al. suggested an oblique tunnel can be created to ensure a longer tunnel [18], while Liu et al. suggested that a 45° tunnel is more likely to avoid tendon injury than a 30° tunnel [14]. However, no studies have yet investigated whether different fibular tunnel directions affect patients' postoperative clinical outcomes. A relevant concept is the GBA angle in ACL reconstruction [27, 32]. Due to friction and impingement between the tendon and the bone tunnel, the resulting shear and compression forces continuously stress the graft, potentially leading to delayed graft maturation [19, 23]. Several previous studies have indicated that the GBA angle is an independent predictor of ligament maturation after ACL reconstruction [5, 20, 26]. Regarding ankle ligament reconstruction, several previous studies have shown that the reconstructed ligament also undergoes a maturation process lasting 1–2 years postoperatively [3, 6]. Therefore, after ankle ligament reconstruction, this mechanism may potentially affect the maturation process of the reconstructed ligament. Our study demonstrates that a 45° tunnel better promotes the maturation of the reconstructed ligament postoperatively, providing further reference for selecting the fibular tunnel direction during ligament reconstruction.

However, no significant differences were observed in the clinical outcomes between the two groups at 12 months and 24 months postoperatively. This aligns with previous findings on ACL reconstruction, in which several studies have reported that GBA has no significant impact on patients' subjective functional outcomes [7, 11, 13]. Besides, this might also be attributed to the relatively small sample size of the study. Several previous studies on ACL reconstruction have identified that MRI SNQ appears to be correlated with certain clinical outcomes, including Tegner score [28], graft failure [21] and re‐laxity [17]. However, these studies all had relatively large sample sizes, ranging from 100 to 300 patients. Therefore, larger‐sample studies are needed to further determine the impact of this imaging finding on patients' clinical outcomes.

There are some limitations to this study. Firstly, as a retrospective study based on complete‐case selection, selection bias may exist. Besides, our study mitigated the influence of other factors on ligament maturation through propensity score matching. However, this also prevented us from comprehensively analysing the effects of different factors on ligament maturation. In addition, the exact CFL treatment differs based on the degree of injury, and such heterogeneity could have potential influence on the study results. Besides, although the possibility was low, angle‐dependent MRI signal changes, including the magic‐angle effect, might provide confounding. Therefore, studies with larger sample sizes, higher levels of evidence and more comprehensive analyses are needed to further investigate potential factors that may influence ATFL maturation after ankle ligament reconstruction.

## CONCLUSION

Compared with a 30° fibular bone tunnel, a 45° tunnel promotes graft healing in CLAI patients following anatomic reconstruction using an autograft.

## AUTHOR CONTRIBUTIONS

All authors contributed to the study conception and design. Conception and design of the study were performed by Dong Jiang. Acquisition of data was performed by Haoxuan Liu, Yuqing Zhao. Analysis of data was performed by Xing Xie, Qinwei Guo and Chen Jiao. Drafting the work was performed by Haoxuan Liu and Yuqing Zhao. Revising it critically for important intellectual content was performed by Yanbin Pi and Dong Jiang. Final approval of the version to be published was performed by Haoxuan Liu, Yuqing Zhao, Tong Su, Xing Xie, Qinwei Guo, Chen Jiao, Yanbin Pi and Dong Jiang.

## FUNDING INFORMATION

Capital Health Research and Development of Special, Grant/Award Number: 2022‐2Z‐40913.

## CONFLICT OF INTEREST STATEMENT

The authors declare no conflicts of interest.

## ETHICS STATEMENT

This study was approved by the IRB Medical Committee of Peking University Third Hospital, IRB00006761‐M20250961. The authors affirm that all participants provided informed consent for publication.

## Data Availability

The datasets used and/or analysed during the current study are available from the corresponding author on reasonable request.
